# PocketPicker: analysis of ligand binding-sites with shape descriptors

**DOI:** 10.1186/1752-153X-1-7

**Published:** 2007-03-13

**Authors:** Martin Weisel, Ewgenij Proschak, Gisbert Schneider

**Affiliations:** 1 Johann Wolfgang Goethe-Universität, Beilstein Endowed Chair for Cheminformatics, Institut für Organische Chemie und Chemische Biologie, Siesmayerstr. 70, D-60323 Frankfurt am Main, Germany

## Abstract

**Background:**

Identification and evaluation of surface binding-pockets and occluded cavities are initial steps in protein structure-based drug design. Characterizing the active site's shape as well as the distribution of surrounding residues plays an important role for a variety of applications such as automated ligand docking or *in situ *modeling. Comparing the shape similarity of binding site geometries of related proteins provides further insights into the mechanisms of ligand binding.

**Results:**

We present PocketPicker, an automated grid-based technique for the prediction of protein binding pockets that specifies the shape of a potential binding-site with regard to its buriedness. The method was applied to a representative set of protein-ligand complexes and their corresponding *apo*-protein structures to evaluate the quality of binding-site predictions. The performance of the pocket detection routine was compared to results achieved with the existing methods CAST, LIGSITE, LIGSITE^cs^, PASS and SURFNET. Success rates PocketPicker were comparable to those of LIGSITE^cs ^and outperformed the other tools. We introduce a descriptor that translates the arrangement of grid points delineating a detected binding-site into a correlation vector. We show that this shape descriptor is suited for comparative analyses of similar binding-site geometry by examining induced-fit phenomena in aldose reductase. This new method uses information derived from calculations of the buriedness of potential binding-sites.

**Conclusion:**

The pocket prediction routine of PocketPicker is a useful tool for identification of potential protein binding-pockets. It produces a convenient representation of binding-site shapes including an intuitive description of their accessibility. The shape-descriptor for automated classification of binding-site geometries can be used as an additional tool complementing elaborate manual inspections.

## Background

Accurate structural information of validated target proteins provides a basis for the design and development of novel therapeutic agents. The increased number of high resolution protein structures available from the RCSB Protein Databank (PDB) [1] has opened new opportunities for structure-based rational drug design [2,3]. Still, the identification of potential protein binding pockets and occluded cavities remains a central issue, as the capability to interact with other proteins or small ligands determines the biological function of a protein. The size and shape of ligand binding sites and the distribution of functional groups in these pockets are of particular interest for the design of selective low-molecular weight ligands. This renders binding-site analysis pivotal for rational drug design, such as ligand docking or *de novo *molecular design. These methods require exact structural information of the binding-site as a starting-point.

**Figure 1:**
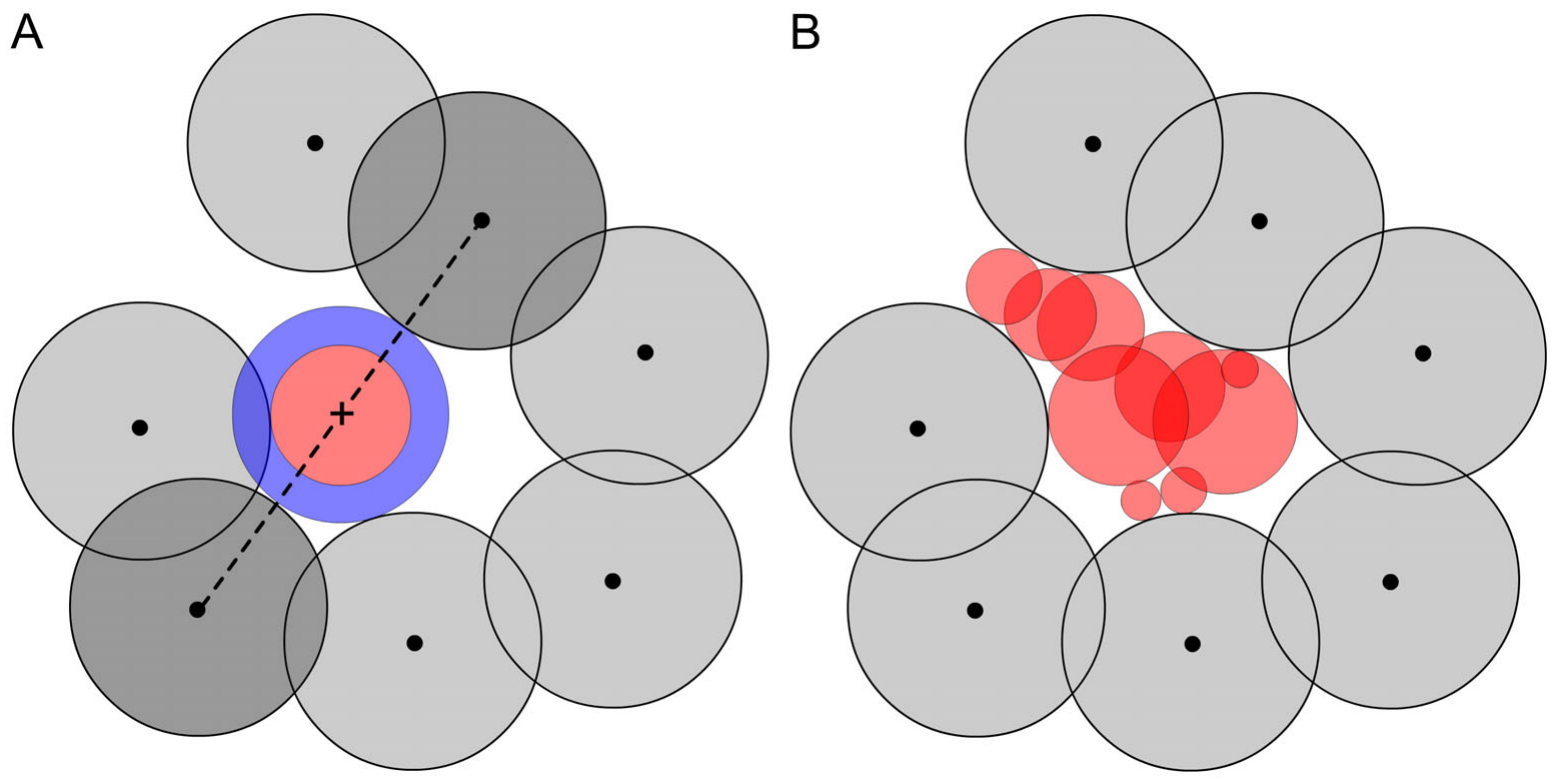
Two-dimensional depiction of the pocket detection process of SURFNET. **A: **An initial gap sphere (blue disc) is placed midway between the van der Waals surfaces of a pair of atoms. The radius of this gap sphere is then reduced until it is not penetrated by any of the neighboring atoms. The resulting final gap sphere is shown in red. **B: **The arrangement of final gap spheres is used to describe the shapes and sizes of protein cavities in SURFNET.

A variety of computational methods already exists for the location of possible ligand binding-sites. Most of these pocket detection algorithms solely rely on geometric criteria to find clefts and surface depressions. Empirical studies show that the actual ligand binding-site usually coincides with the largest pocket of a protein's surface [4,5]. The program SURFNET [6] successfully predicted the ligand binding-site as the biggest pocket in 83% of the cases on a test set of 67 single-chain enzymes [7]. SURFNET identifies voids between two or more molecules as well as internal cavities and pockets by fitting virtual spheres into the solvent-accessible space between protein atoms. So-called "initial gap spheres" are placed midway between the van-der-Waals surfaces of two atoms and scaled down when penetrated by neighboring atoms. All remaining gap spheres exceeding a minimal predefined radius (default is 1.0 Å) are denoted as "final spheres" and used to define surface pockets and cavities (Figure 1).

**Figure 2:**
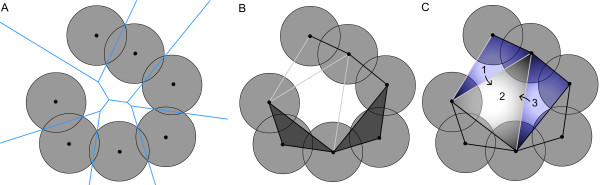
Illustration of the alpha shape theory and discrete-flow method used in CAST. **A: **Two-dimensional depiction of pocket atoms represented as disks of uniform radii. The blue lines show the Voronoi diagram for the pocket atoms. **B: **The seven bordering lines running through the atom centers represent the convex hull, which is triangulated into Delaunay triangles using information of the Voronoi diagram. The "alpha shape" or "dual complex" is defined by the shaded triangles and the black lines. Three "empty triangles" having at least one grey bordering line are shown. **C: **Two obtuse empty triangles (1, 3) are assigned to the obtuse triangle (2) by the discrete-flow method.

The program CAST [8,9] uses an approach based on alpha shapes [10,11] and triangulations of complex shapes. This method makes use of the concepts of Voronoi diagrams [12] and Delaunay [13] triangulations. The pocket prediction process of CAST specifies the calculation of the so-called "dual complex" (or alpha shape) and is summed up for a simplified two-dimensional depiction of binding site atoms (Figure 2). The procedure includes the calculation of the Voronoi diagram which consists of Voronoi cells (Figure 2A). Each Voronoi cell contains one protein atom and controls all spatial points that are closest to the respectively considered atom. The Voronoi diagram is mathematically equivalent to the Delaunay triangulation of the complex hull drawn around the protein atom centers (Figure 2B). The Delaunay triangulation can be obtained directly from the Voronoi diagram. Therefore a line is drawn across every Voronoi edge separating two Voronoi cells connecting the two corresponding atoms centers. For each Voronoi vertex where three Voronoi cells meet, a Delaunay triangle is placed connecting the three atom centers of the considered cells. To obtain the dual complex, Voronoi edges and vertices are disregarded in the triangulation, if they are situated completely or in part outside of the molecule (Figure 2B, grey lines). A triangle with one ore more omitted edges is denoted as "empty". Neighboring empty triangles are combined in the "disrete-flow" method to outline continuous voids in the protein surface. In the course of this process an obtuse empty triangle flows to its neighboring triangle, whereas acute empty triangles act as sinks to collect the flow of neighboring triangles (Figure 2C). CAST was tested on 51 of 67 monomeric complexes used for SURFNET [6] and achieved a success rate of 74%.

**Figure 3:**
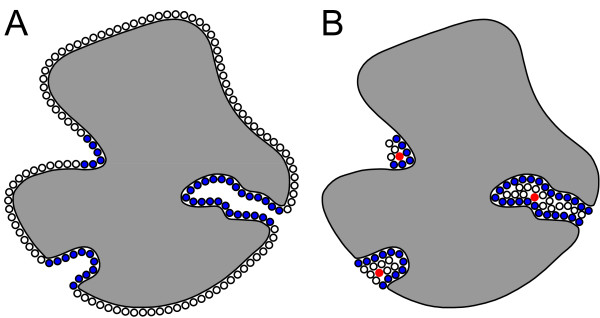
Placement of spheres for a two-dimensional molecule in PASS. **A: **The entire surface of the molecule is coated with virtual spheres and an initial layer of spheres residing in buried parts of the protein is specified (blue shaded circles). **B: **Additional layers are attached onto the initial layer in an iterative process and active site points (red disks) are exposed for potential binding pockets.

PASS [14] (Putative Active Sites with Spheres) uses an iterative placing of probe spheres to identify surface concavities. An initial layer of probe spheres coating the entire protein surface is created in the first step (Figure 3). For each probe sphere a "burial count" is calculated which gives the number of protein atoms within a preset radius of 8 Å. This measurement is used to identify probe spheres located in protein surface pockets and cavities. Probe spheres residing in convex parts of the surface are omitted from further calculations. Additional layers are then accreted to the remaining probe set to completely fill protein cavities with probe spheres. A "probe weight" is calculated for each probe sphere of the final set comprising the burial count and the number of neighboring probe spheres. Finally, a small number of "active site points" (ASPs) is selected to represent the centers of potential binding pockets. ASPs are identified by picking central probes from regions containing many spheres of high burial counts. Putative binding sites are defined by keeping a reduced set of ASPs separated by a minimum threshold of 8 Å. Pockets are ranked by the probe weights of their corresponding ASPs. PASS yielded correct predictions of 63% on a set of 30 complexed structures and 60% for a test set of 20 *apo*-protein structures.

**Figure 4:**
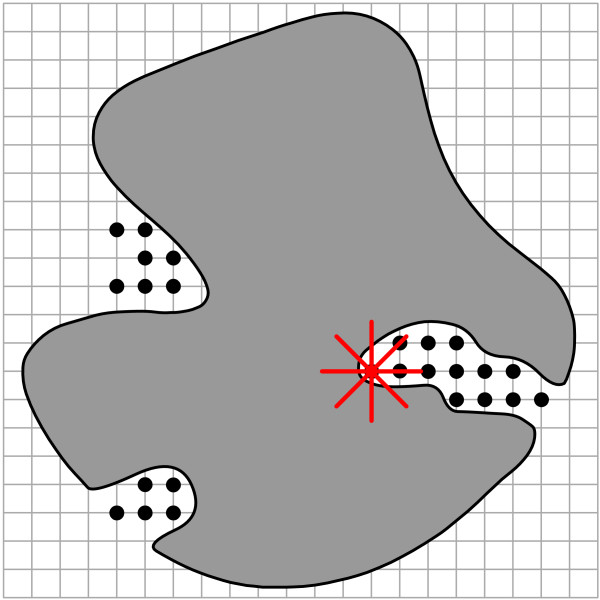
Pocket detection method used in POCKET, LIGSITE and its derivatives. Grid probes are installed at the edges of an artificial grid generated around the protein (shaded area). A scanning process is applied to detect protein-solvent-protein events (POCKET and LIGSITE) or surface-solvent-surface events (LIGSITE^cs ^and LIGSITE^csc^).

Another pocket detection method is POCKET [15]. This algorithm operates on a rectangular grid, which is constructed around the protein and denotes grid points as either solvent-accessible or inaccessible to the solvent. The program searches for cavities by scanning along the *x*-, *y*- and *z*-axes to locate groups of solvent-accessible grid points that are enclosed by grid points not accessible to solvent on both sides (Figure 4). Such arrangements were denoted as PSP events (protein-solvent-protein). Results of POCKET may be unsatisfying as pockets with an orientation of 45° to the orthogonal axes will not be properly detected or even be totally ignored. To compensate for this deficiency LIGSITE [16] was developed as an extension to POCKET. In this approach the scanning process was extended to the four cubic diagonals so that a proper pocket prediction became possible, which is independent from the orientation of the protein in the grid. LIGSITE^cs ^and LIGSITE^csc ^were introduced as enhanced implementations of the original LIGSITE [16] algorithm and resulted in improved pocket prediction results [17]. LIGSITE^cs ^(cs = Conolly Surface) differs from the original LIGSITE [16] method by capturing surface-solvent-surface events using the protein's Conolly surface instead of detecting protein-solvent-protein events. LIGSITE^csc ^(csc = Conolly Surface and Conservation) performs a re-ranking of the top-three predicted pockets by the degree of conservation of the closest surface residues. The average conservation of the residues within 8 Å of the center of a predicted pocket is used as a conservation score applied for re-ranking. Note that LIGSITE^csc ^is not a purely geometric approach to pocket prediction as it considers conservation scores obtained from the ConSurf-HSSP [18] database as an additional source of information. A refinement of the predictions made by SURFNET [6] using conservation scores for re-ranking is also available from a subsequent recent study [19].

Further algorithms exclusively operating on geometric criteria are Cavity Search [20], VOIDOO [21], APROPOS [22], and Travel Depth [23]. DrugSite [24] and PocketFinder [25] evaluate shape and physicochemical properties for identification of ligand binding envelopes. An energy-based method for protein pocket detection is Q-SiteFinder [26], which uses the interaction energy between the protein and a van der Waals probe to detect energetically favorable binding sites.

In this study, we present a new geometric pocket prediction method that translates the form and accessibility of identified binding-sites into correlation vectors for rapid pocket comparisons. A similar approach was pursued by Stahl *et al*. with the aim to classify matrix metalloproteinase active sites [27]. The pocket detection routine is based on a regular rectangular grid and employs a sophisticated scanning process to locate protein surface depressions. The scanning procedure comprises the calculation of "buriedness" of probe points installed in the grid to determine their atom environment. The buriedness of grid points is interpreted as a pocket accessibility index. The enhanced information content of both the buriedness and the shape of a predicted binding pocket is summarized in a shape descriptor. This descriptor has been designed to conduct automated comparisons between different binding-site conformations. The essential steps of our method can be summed up as follows:

(1) Calculation of buriedness values of grid probes installed in areas closely above the protein surface.

(2) Clustering of adjoining grid probes indicating buried regions of the structure to find potential binding-sites.

(3) Preparation of shape descriptors to enable comparisons of different pocket shapes.

## Materials and computational methods

### Protein data collection

To evaluate the accuracy of binding site predictions performed by PocketPicker we used a test set comprising 48 ligand-receptor complexes from the RCSB Protein Database (PDB [1]) as well as their corresponding 48 unbound *apo*-forms. This test set was presented in a previous study [17] to compare success rates of pocket predictions by the programs CAST, PASS, SURFNET, LIGSITE, LIGSITE^cs^, and LIGSITE^csc^. We used this protein collection to validate the predictions made by PocketPicker compared to the findings of these algorithms. All protein structures were downloaded from the RCSB PDB database [1], and ligands denoted with the HET (heteroatom) identifier were removed from each PDB-file prior to computations. Binding site predictions were carried out for monomeric structures (results for protein multimers are provided as additional files [see Additional files 1, 2]). Unbound structures were aligned with the corresponding complex using the "align" command of PyMOL [28]. Structural alignments were performed to compare active site predictions for the unbound structures with the actual binding pocket given by the protein-ligand complex.

The capability of comparing induced-fit phenomena with the proposed shape descriptor was tested on a set of 13 aldose reductase crystal structures discussed by Sotriffer and coworkers [29]. This selection contained nine structures of human aldose reductase: 1ads, 1el3, 1iei, 1us0, 2acq, 2acr, 2acs, the Tyr48His mutant 2acu, and the Cys298Ala/Trp219Tyr double mutant 1az1. Additional four structures were from the porcine enzyme and carried one mutation each: 1ah0, 1ah3, 1ah4, and 1eko. The crystal structure of 1us0 with an ultrahigh resolution of 0.66 Å served as a reference. All selected structures shared a sequence identity of ≥ 85% with the reference and had resolution of at least 2.5 Å. Coordinates of 1ah0, 1ah3, 1ah4 and 1eko were rotated -45° around the *z*-axis to meet the orientation of the other aldose reductase structures. Pocket predictions were performed for structures in complex with the cofactor NADPH or NADP^+^. All other ligands were removed prior to computation.

### Strategy for identification of surface pockets and cavities

**Figure 5:**
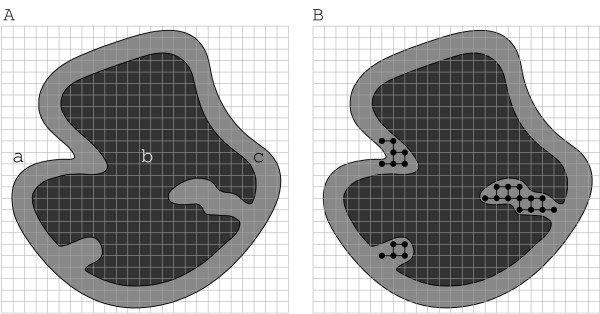
Schematic view of the pocket detection process of PocketPicker. **A: **Grid points located far off the protein (a) or hidden under the surface (b) are excluded from calculations. Buriedness values are calculated solely for grid points close to the protein surface (c). **B: **Grid probes indicating surface depressions are collected in clusters.

A rectangular grid with 1 Å mesh size is generated around the protein, adjusted to its spatial extent. The pocket detection routine is focused on grid points that are located closely above the protein surface: grid points that exceed a maximal distance of 4.5 Å to the closest protein atom or are situated under the protein surface are excluded from further calculations (Figure 5a). Note that these areas can be omitted from further investigation, since they are not relevant for pocket detection. Probes are attached to the remaining grid points to examine their accessibility on the protein surface.

The buriedness value indicates whether a grid point is situated next to a convex part of the surface or locates in a less accessible part of the surface. This information can be used for the identification of clefts and surface concavities: A straightforward clustering algorithm is applied to combine neighboring grid points with an appropriate buriedness-index into disjoint groups highlighting those parts of the grid located in less accessible parts of the protein surface (Figure 5b). Cavities and pockets identified in this manner are afterwards sorted by the number of the consisting grid points to specify the largest existing protein concavity.

### Calculation of buriedness

**Figure 6:**
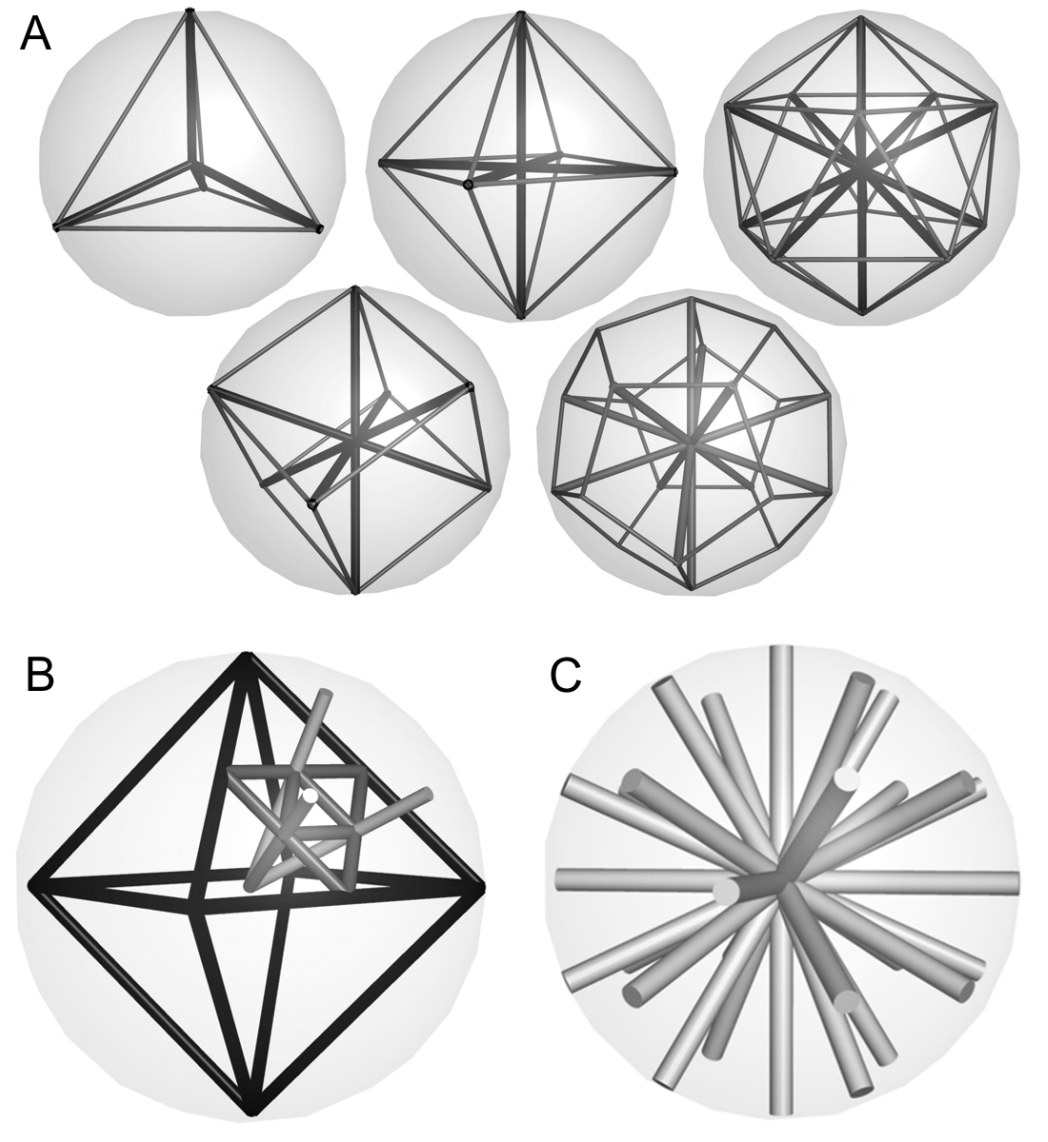
Triangulations of the sphere. **A: **The five Platonic bodies offer a symmetric decomposition of the sphere, but only the tetrahedron, the octahedron and the icosahedron (upper row) describe an exact spherical equidistribution of vectors. **B: **Triangulation of the octahedron was used to arrange additional vectors on the sphere. **C: **Distribution of 30 search rays obtained from octahedron triangulation.

The buriedness-index is calculated by investigating the molecular environment of a grid probe in an elaborate scanning process: Scans are being performed along 30 directions that are approximately equally distributed around a grid probe. The optimal distribution of vectors in three-dimensional space is not a trivial problem and resembles the task of equally distributing points on a sphere [30]. In fact, there are only three completely symmetric arrangements of points (*n *> 2) on the sphere: The vertices of the tetrahedron, the octahedron and the icosahedron are equally distributed [31] on a commemorated sphere (Figure 6a).

We use a series of triangulations to subdivide the eight faces of the octahedron in order to arrange three additional vectors on each face (Figure 6b). These newly added vectors are elongated toward the surface of a virtual sphere to adopt the length of the primary vectors of the octahedron running along the Cartesian axes (Figure 6c). The 30 vectors created in this manner can be reflected in the *x*, *z*-plane which is required for a subsequent part of the computation.

The accessibility of a grid probe is calculated by scanning the molecular surrounding along 30 search rays of length 10 Å and width 0.9 Å. Whenever a protein atom is encountered within the dimensions of a search ray, the buriedness-index of the probe is increased by one and the next direction vector is regarded. As a result, the calculated indices range from 0 to 30 indicating a growing buriedness of the probe in a protein. The clustering of grid probes for pocket identification is restricted to those probes with buriedness-indices ranging from 16 to 26.

Direction vectors u→
 MathType@MTEF@5@5@+=feaafiart1ev1aaatCvAUfKttLearuWrP9MDH5MBPbIqV92AaeXatLxBI9gBaebbnrfifHhDYfgasaacH8akY=wiFfYdH8Gipec8Eeeu0xXdbba9frFj0=OqFfea0dXdd9vqai=hGuQ8kuc9pgc9s8qqaq=dirpe0xb9q8qiLsFr0=vr0=vr0dc8meaabaqaciaacaGaaeqabaqabeGadaaakeaacuWG1bqDgaWcaaaa@2E31@ are aligned along 30 straight lines *G *arranged by octahedron triangulation and scaled to the length of one. Search rays scanning the molecular environment of a grid probe *P *(represented by vector p→
 MathType@MTEF@5@5@+=feaafiart1ev1aaatCvAUfKttLearuWrP9MDH5MBPbIqV92AaeXatLxBI9gBaebbnrfifHhDYfgasaacH8akY=wiFfYdH8Gipec8Eeeu0xXdbba9frFj0=OqFfea0dXdd9vqai=hGuQ8kuc9pgc9s8qqaq=dirpe0xb9q8qiLsFr0=vr0=vr0dc8meaabaqaciaacaGaaeqabaqabeGadaaakeaacuWGWbaCgaWcaaaa@2E27@) are arranged along the direction vectors and scaled to the proposed dimensions. A neighboring protein atom *Q *(q→
 MathType@MTEF@5@5@+=feaafiart1ev1aaatCvAUfKttLearuWrP9MDH5MBPbIqV92AaeXatLxBI9gBaebbnrfifHhDYfgasaacH8akY=wiFfYdH8Gipec8Eeeu0xXdbba9frFj0=OqFfea0dXdd9vqai=hGuQ8kuc9pgc9s8qqaq=dirpe0xb9q8qiLsFr0=vr0=vr0dc8meaabaqaciaacaGaaeqabaqabeGadaaakeaacuWGXbqCgaWcaaaa@2E29@) is detected during scanning when the length of its orthogonal projection *d *onto the actual direction vector does not exceed the preset width of the search ray.

d=|(q→−p→)×u→|, if|u→|=1
 MathType@MTEF@5@5@+=feaafiart1ev1aaatCvAUfKttLearuWrP9MDH5MBPbIqV92AaeXatLxBI9gBaebbnrfifHhDYfgasaacH8akY=wiFfYdH8Gipec8Eeeu0xXdbba9frFj0=OqFfea0dXdd9vqai=hGuQ8kuc9pgc9s8qqaq=dirpe0xb9q8qiLsFr0=vr0=vr0dc8meaabaqaciaacaGaaeqabaqabeGadaaakeaacqWGKbazcqGH9aqpdaabdaqaaiabcIcaOiqbdghaXzaalaGaeyOeI0IafmiCaaNbaSaacqGGPaqkcqGHxdaTcuWG1bqDgaWcaaGaay5bSlaawIa7aiabcYcaSiabbccaGiabbMgaPjabbAgaMnaaemaabaGafmyDauNbaSaaaiaawEa7caGLiWoacqGH9aqpcqaIXaqmaaa@4648@

The projected point *X *has to reside within the length of the search ray. The distance between *X *and the actual grid point *P *can be determined as the length of the direction vector u→
 MathType@MTEF@5@5@+=feaafiart1ev1aaatCvAUfKttLearuWrP9MDH5MBPbIqV92AaeXatLxBI9gBaebbnrfifHhDYfgasaacH8akY=wiFfYdH8Gipec8Eeeu0xXdbba9frFj0=OqFfea0dXdd9vqai=hGuQ8kuc9pgc9s8qqaq=dirpe0xb9q8qiLsFr0=vr0=vr0dc8meaabaqaciaacaGaaeqabaqabeGadaaakeaacuWG1bqDgaWcaaaa@2E31@ scaled by *t*.

Factor *t *was calculated as follows:

t=(q→−p→)⋅u→, if|u→|=1
 MathType@MTEF@5@5@+=feaafiart1ev1aaatCvAUfKttLearuWrP9MDH5MBPbIqV92AaeXatLxBI9gBaebbnrfifHhDYfgasaacH8akY=wiFfYdH8Gipec8Eeeu0xXdbba9frFj0=OqFfea0dXdd9vqai=hGuQ8kuc9pgc9s8qqaq=dirpe0xb9q8qiLsFr0=vr0=vr0dc8meaabaqaciaacaGaaeqabaqabeGadaaakeaacqWG0baDcqGH9aqpcqGGOaakcuWGXbqCgaWcaiabgkHiTiqbdchaWzaalaGaeiykaKIaeyyXICTafmyDauNbaSaacqGGSaalcqqGGaaicqqGPbqAcqqGMbGzdaabdaqaaiqbdwha1zaalaaacaGLhWUaayjcSdGaeyypa0JaeGymaedaaa@4379@

**Figure 7:**
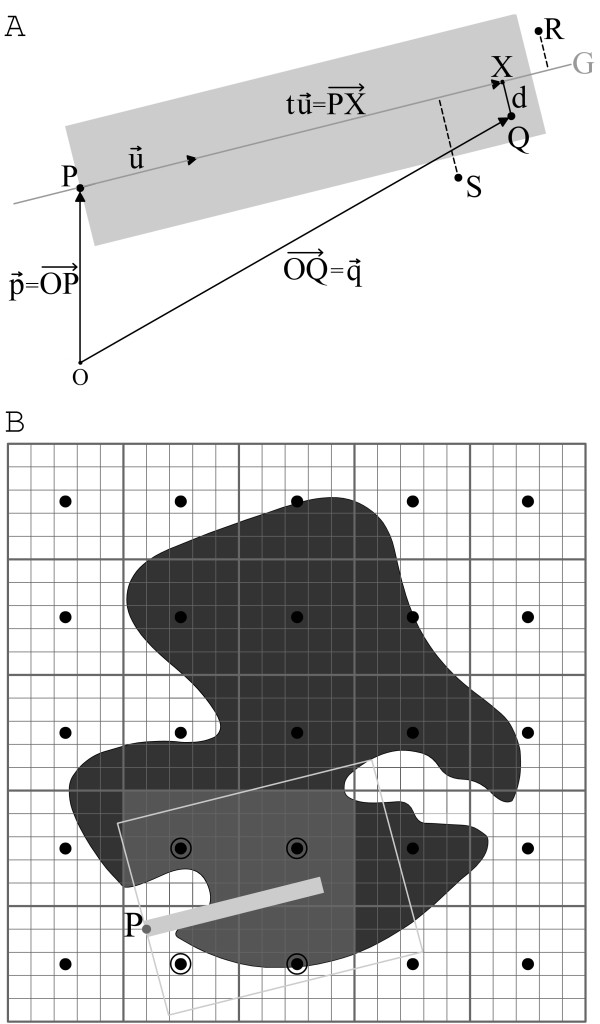
Calculation of the buriedness-index of a grid point *P*. **A: **A search ray (grey plane) scans the room for atoms. Atom *Q *is detected, since it is located within the dimensions of the search vector. Atoms *R *and *S *are not detected, since they are not covered by the search vector. **B: **Distance calculations are restricted to areas controlled by neighboring centroids (encircled). Neighboring centroids are identified by scanning an extended search space (grey border).

The scanning process is summarized in Figure 7a. Position vectors of all atoms and grid points use the Cartesian origin *O *as their reference point.

In order to avoid distance calculations to all protein atoms, the search grid is subdivided into smaller cuboidal compartments of same size, and represented by centroids denoting the geometric center of a cuboid. In a first step, neighboring centroids are detected in an extended search radius along the actual direction vector. Distance calculations are then performed solely to protein atoms assigned to the cuboids of the regarded centroids (Figure 7b).

### Comparison of pocket shapes

A descriptor was designed to describe the shape of a pocket with respect to the buriedness of the site. Grid probes were grouped into six categories A, B, C, D, E, F holding grid point coordinates with ascending buriedness values: A: 15–16, B: 17–18, C: 19–20, D: 21–22, E: 23–24, F: 25–26. The shape descriptor was developed to record the appearance of distances between pairs of these categories. Distances were staggered in 20 distance bins covering ranges up to 20 Å for 21 possible combinations of the six categories. Pocket shapes were compared with respect to their buriedness by calculating the Euclidean distance *d *between the resulting 420-dimensional shape descriptors of two molecules, *r *and *s*:

d=∑i=1420(ri−si)2
 MathType@MTEF@5@5@+=feaafiart1ev1aaatCvAUfKttLearuWrP9MDH5MBPbIqV92AaeXatLxBI9gBaebbnrfifHhDYfgasaacH8akY=wiFfYdH8Gipec8Eeeu0xXdbba9frFj0=OqFfea0dXdd9vqai=hGuQ8kuc9pgc9s8qqaq=dirpe0xb9q8qiLsFr0=vr0=vr0dc8meaabaqaciaacaGaaeqabaqabeGadaaakeaacqWGKbazcqGH9aqpdaGcaaqaamaaqahabaGaeiikaGIaemOCai3aaSbaaSqaaiabdMgaPbqabaGccqGHsislcqWGZbWCdaWgaaWcbaGaemyAaKgabeaakiabcMcaPmaaCaaaleqabaGaeGOmaidaaaqaaiabdMgaPjabg2da9iabigdaXaqaaiabisda0iabikdaYiabicdaWaqdcqGHris5aaWcbeaaaaa@4138@

## Results

### Evaluation of pocket prediction

To assess the quality of PocketPicker's binding-site predictions we refer to an evaluation method already applied in previous studies [14,17]. Thus, we define a prediction to be a hit, if the geometric center of the presumed pocket lies within 4 Å to any atom of the ligand. Predictions that do not meet this criterion were excluded for calculation of prediction success rates.

The search routine of PocketPicker was evaluated on a test set of 48 protein-ligand complexes and the respective *apo*-structures. Evaluation of pocket predictions for uncomplexed structures is of special interest for geometric search algorithms as the absence of a pocket-inducing ligand might complicate pocket identifications.

Success rates of pocket predictions were compared to the findings of other prediction methods presented in a study published by Huang and Schröder [17]. The test set was compiled as described therein to allow for a comparison of results. Note that slight discrepancies to the original test set cannot be ruled out due to differences in data preparation.

Pocket prediction results were divided into different categories for quality assessment: Correct predictions were termed "TOP1-hits" whereas "TOP3-hits" are predictions where the respective ligand is found within the three largest predicted pockets. Success rates of pocket predictions are summarized in Table 2. Prediction results are given for the proposed methods and their performance on the dataset of 48 bound/unbound structures indicating TOP1- and TOP3-hits.

**Table 1:** Collection of 48 complexes and their corresponding *apo*-forms to evaluate pocket prediction results.

**Complex**	**Unbound**	**Protein Description**	**Pocket Ligand^1^**	**Other Ligands^2^**
1bid	3tms	Thymidylate synthase	UMP	CBX
1cdo	8adh	Alcohol dehydrogenase	NAD	zn
1dwd	1hxf	Alpha thrombin	MID	chains i, l
1fbp	2fbp	Fructose 1,6-bisphosphatase	AMP	F6P, mg
1gca	1gcg	Glucose/galactose-binding protein	GAL	ca
1hew	1hel	Hen egg white lysozyme	NAG	-
1hyt	1npc	Thermolysin	BZS	DMS, ca, zn
1inc	1esa	Elastase	ICL	ca, so4
1rbp	1brq	Retinol binding protein	RTL	-
1rob	8rat	Ribonuclease A	C2P	-
1stp	1swb	Streptavidin	BTN	-
1ulb	1ula	Purine nucleoside phosphorylase	GUN	so4
2ifb	1ifb	Fatty acid binding protein	PLM	-
3ptb	3ptn	Beta trypsin	BEN	ca
2ypi	1ypi	Triose phosphate isomerase	PGA	-
4dfr	5dfr	Dihydrofolate reductase	MTX	ca, cl
4phv	3phv	HIV 1 protease	VAC	-
5cna	2ctv	Concanavalin A	MMA	ca, cl, mn
7cpa	5cpa	Carboxypeptidase A	FVF	zn
1a6w	1a6u	B1-8 FV fragment	NIP	-
1acj	1qif	Acetylcholinesterase	THA	-
1apu	3app	Penicillopepsin	[IVA-VAL-VAL-STA-OET]	MAN
1blh	1djb	Beta-lactamase	FOS	-
1byb	1bya	Beta amylase	GLC	so4
1hfc	1cge	Fibroblast collagenase	HAP	ca, zn
1ida	1hsi	HIV 2 protease	[QND-VAL-HPB-PPL-PY2]	-
1igj	1a4j	Immunoglobulin	DGX	chain y
1imb	1ime	Inositol monophosphatase	LIP	gd
1ivd	1nna	Hydrolase	ST1	FUC, NAG, MAN, ca
1mrg	1ahc	Alpha momorcharin	ADN	-
1mtw	2tga	Trypsin	DX9	ca
1okm	4ca2	Carbonic anhydrase II	SAB	hg, zn
1pdz	1pdy	Enolase	PGA	ace, mn
1phd	1phc	Camphor 5-monoxygenase	PIM	HEM
1pso	1psn	Pepsin 3a	[IVA-VAL-VAL-STA-ALA-STA]	-
1qpe	3lck	Lck kinase	PP2	PTR, so4
1rne	1bbs	Renin	C60	NAG
1snc	1stn	Staphylococcal nuclease	PTP	ca
1srf	1pts	Streptavidin	MTB	-
2ctc	2ctb	Carboxypeptidase A	LOF	zn
2h4n	2cba	Carbonic anhydrase II	AZM	zn
2pk4	1krn	Plasminogen kringle	ACA	-
2sim	2sil	Sialidase	DAN	-
2tmn	1l3f	Thermolysin	[PHO-LEU-NH2]	ca, zn
3gch	1chg	Gamma chymotrypsin	CIN	-
3mth	6ins	Methylparaben insulin	MPB	cl, zn
5p2p	3p2p	Phosphilipase	DHG	ca
6rsa	7rat	Ribonuclease A	UVC	dod

^1^Considered ligand defining the active site. Brackets indicate ligands composed of multiple residues or fragments.

^2^Additional ligands and ions were removed prior to computations.

**Table 2:** Comparison of success rates for 48 complexed und 48 unbound protein structures.

	**Top 1**		**Top 3**	
	
	**Unbound**	**Bound**	**Unbound**	**Bound**
PocketPicker	69	72	85	85
LIGSITE^cs^	60	69	77	87
LIGSITE	58	69	75	87
CAST	58	67	75	83
PASS	60	63	71	81
SURFNET	52	54	75	78
LIGSITE^csc1^	71	79	-	-

^1^Results of LIGSITE^csc ^are given for the sake of completeness. Note that the comparability to the other methods findings is limited, due to the fact that LIGSITE^csc ^is not a purely geometric approach.

PocketPicker outperformed CAST, PASS and SURFNET, and showed advantages over LIGSITE and LIGSITE^cs^. These two programs only showed slightly better success rates for the TOP3-hits on bound protein structures. Results of pocket predictions on the two test data sets are provided for PocketPicker in Table 3, indicating the rank of the proposed binding site and the distance between the pocket center and the nearest ligand atom. The summary of results obtained with LIGSITE^csc^, LIGSITE, PASS, SURFNET and CAST is available in the work of Huang and Schröder [17].

**Table 3:** Prediction success of PocketPicker on 48 bound and unbound structures.

**Complex**	**Hits^1^**	**D_near_/Å)^2^**	**Unbound**	**Hits^1^**	**D_near_/Å)^2^**
1bid	1	2.7	3tms	1	2.3
1cdo	1	2.3	8adh	1	1.9
1dwd	1	0.5	1hxf	1	0.3
1fbp	1	1.3	2fbp	4	1.2
1gca	1	2.4	1gcg	1	1.4
1hew	1	1.4	1hel	1	1.2
1hyt	1	1.3	1npc	1	1.7
1inc	1	0.5	1esa	(1)	4.1
1rbp	1	0.8	1brq	1	1.0
1rob	1	1.7	8rat	1	1.9
1stp	1	2.4	1swb	1	1.0
1ulb	1	1.0	1ula	(1)	4.4
2ifb	1	1.7	1ifb	1	2.5
3ptb	1	0.4	3ptn	3	1.0
2ypi	5	1.0	1ypi	(1)	4.8
4dfr	(1)	7.8	5dfr	1	1.8
4phv	2	2.7	3phv	2	3.5
5cna	-	-	2ctv	-	-
7cpa	1	1.0	5cpa	1	1.1
1a6w	2	1.4	1a6u	3	1.2
1acj	1	0.8	1qif	2	1.2
1apu	1	0.6	3app	1	0.5
1blh	1	1.0	1djb	1	0.8
1byb	1	3.3	1bya	1	3.6
1hfc	1	1.2	1cge	1	1.0
1ida	1	1.5	1hsi	1	3.2
1igj	4	1.6	1a4j	3	1.4
1imb	(1)	5.5	1ime	1	3.4
1ivd	2	1.7	1nna	1	1.5
1mrg	(1)	5.8	1ahc	(1)	5.2
1mtw	2	0.8	2tga	4	0.6
1okm	2	1.2	4ca2	1	1.6
1pdz	1	2.2	1pdy	1	2.7
1phd	1	1.1	1phc	1	0.9
1pso	1	0.4	1psn	1	1.1
1qpe	1	0.9	3lck	1	1.1
1rne	1	1.7	1bbs	1	0.7
1snc	1	2.1	1stn	1	0.3
1srf	1	0.5	1pts	1	0.6
2ctc	1	1.2	2ctb	1	1.5
2h4n	1	0.8	2cba	1	2.1
2pk4	2	0.7	1krn	1	0.7
2sim	2	0.6	2sil	2	0.4
2tmn	1	1.3	1l3f	1	1.1
3gch	1	0.8	1chg	2	1.5
3mth	2	0.8	6ins	2	1.3
5p2p	1	1.0	3p2p	1	0.8
6rsa	1	3.0	7rat	1	0.9

^1^Rank of pocket centers within 4 Å of the considered ligand (brackets indicate hits exceeding the 4 Å criterion). Only the best hit is shown. Dashes indicate that the actual binding site is not found within the five largest predicted pockets.

^2^Distance from the geometric pocket center to the nearest atom in the ligand.

### Analysis of induced fit phenomena

The capability of the proposed shape descriptor to detect conformational similarities in pocket shapes of aldose reductase structures was assessed with respect to the structural analyses presented by Sotriffer and coworkers [29]. Four distinct binding-sites conformations were distinguished by visual inspection, named after the respective ligand characterizing a separate class of pocket shapes: the "IDD594"-conformation, the "holo"-conformation (the cofactor-bound, but ligand-free conformation), the "tolrestat"-conformation, and the "zenarestat"-conformation. In our study, we used these terms to address different classes of structural conformations caused by induced fit phenomena upon ligand binding.

**Figure 8:**
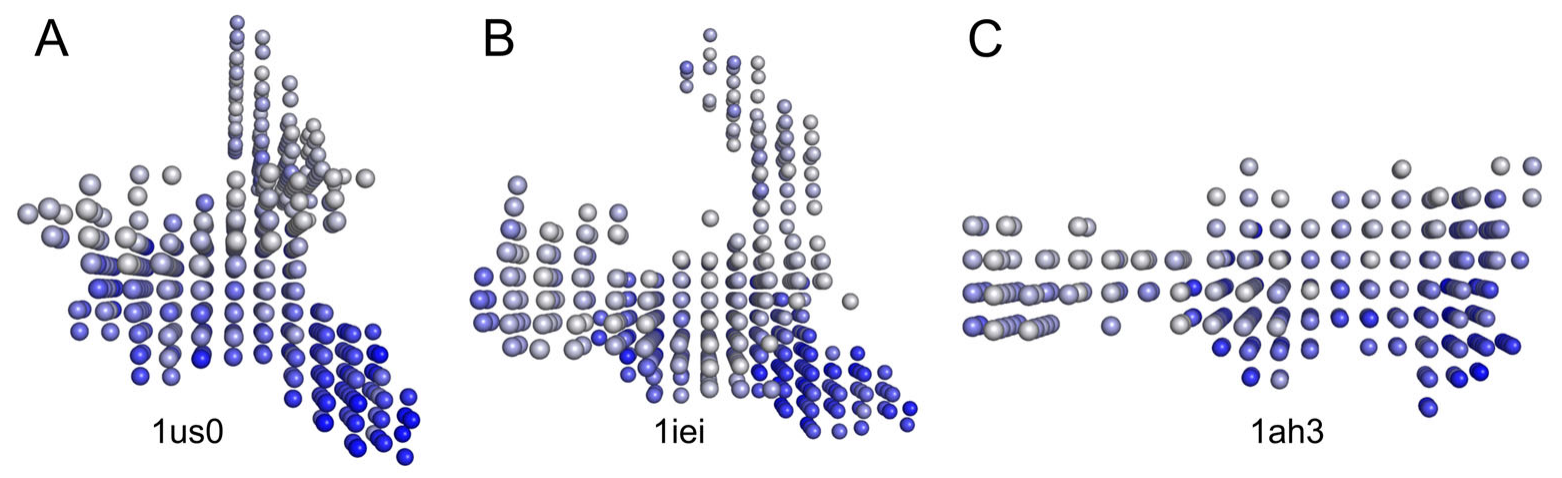
Shapes of pocket conformations induced by IDD594 (**A**), zenarestat (**B**) and tolrestat (**C**). Binding sites are given in PocketPicker representation with darker spheres indicating greater buriedness.

The complex formed between aldose reductase and the potent inhibitor (*IC*_50 _= 30 nM) IDD594 (PDB-ID 1us0 [32]) represents its own conformational class in the selected set of structures (Figure 8a). A structural similarity to the zenarestat-conformation (1iei, Figure 8b) was revealed using the calculated shape descriptors. Of all the structures observed in this study, the shape descriptor of the 1iei binding-site showed the smallest Euclidean distance to the IDD594-conformation (Table 4). The binding modes of zenarestat and IDD594 are reported as fairly similar [29], which could explain the structural similarities of the binding-site conformations.

**Table 4:** Euclidean distances between pocket shape descriptors. Distances of very similar pocket shapes (*d *< 2000) are highlighted.

	**1ah0**	**1ah3**	**1ah4**	**1eko**	**1el3**	**1iei**	**1us0**	**2acq**	**2acr**	**2acs**	**2acu**
**1ads**	3735	6480	3762	3453	**1527**	3935	4773	2890	4263	2099	4966
**1ah0**		3749	**1758**	2528	3093	2943	2787	2516	2471	2152	3157
**1ah3**			3608	4116	5620	3873	3168	4739	3480	4939	3361
**1ah4**				**1727**	2967	3125	3130	2461	2254	2706	2512
**1eko**					2452	2972	3046	2693	2910	2884	2752
**1el3**						3414	4084	2499	3664	**1892**	4033
**1iei**							2415	3314	3401	2833	3863
**1us0**								3147	2894	3549	2940
**2acq**									**1946**	2267	2617
**2acr**										3058	**1862**
**2acs**											4019

The tolrestat-complex (1ah3, Figure 8c) depicts a further binding-site conformation that is substantially different to the other pocket geometries discussed here [29]. This fact is again recognized by our shape descriptor showing pronounced Euclidean distances to the remaining structures (Table 4).

**Figure 9:**
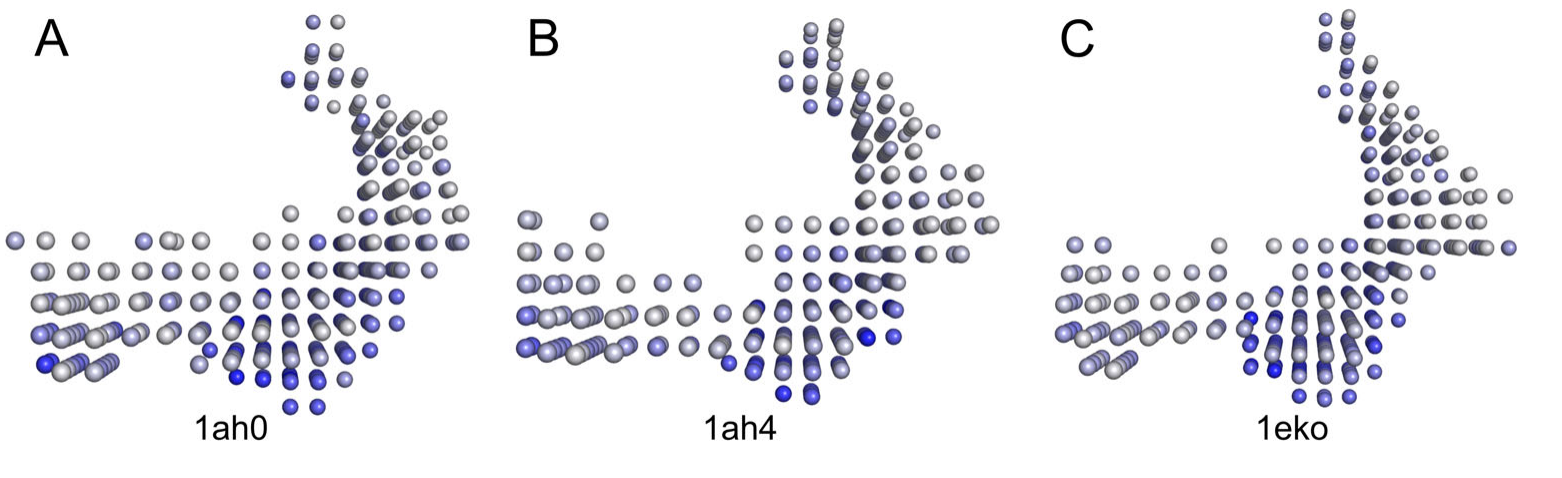
Pocket shapes of the *holo*-conformation subset 1ah0/1ah4/1eko.

The majority of the binding-site conformations was assigned to the *holo*-conformation (1ads, 1ah0, 1ah4, 1az1, 1eko, 1el3, 2acq, 2acr, 2acs, 2acu) with three structures (1ah0, 1ah4, 1eko) forming a subset with only minor differences to the standard *holo*-conformation [29] (Figure 9).

**Figure 10:**
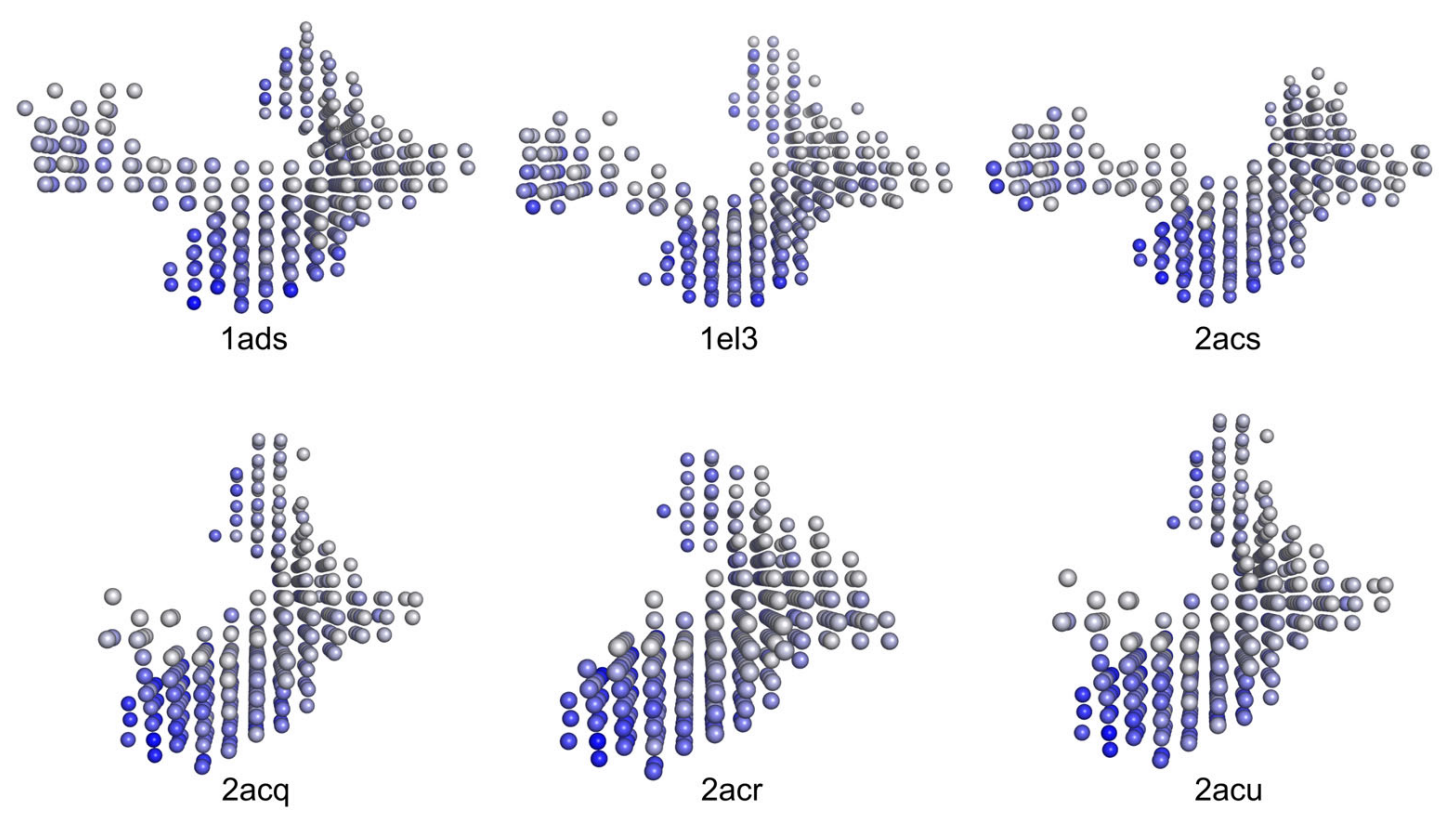
Pocket shapes of the *holo*-conformation with 1ads/1el3/2acs and 2acq/2acr/2acu forming similar subsets.

The conformational similarity of the active sites of this subgroup is reflected in the calculated shape descriptors: Taking 1ah4 as a reference, the remaining members of this subset are correctly identified as the two entries with the lowest Euclidean distance (Table 4). Following this strategy, we were able to identify additional two subsets within the *holo*-conformation set: Considering 1el3 and 2acr as references presenting two strikingly similar entries (Euclidean distance < 2000), we detected the subsets {1ads, 1el3, 2acs} and {2acq, 2acr, 2acu}. Structural similarity in binding-site conformations can be comprehended by the visual information offered by PocketPicker (Figure 10).

PocketPicker was able to correctly predict the active sites of all aldose reductase structures tested, with the exception of the binding-site geometry of 1az1, which shows major differences compared to the *holo*-conformation.

## Discussion

The pocket identification algorithm follows the concept of grid-based detection methods. The usage of an increased number of 30 scanning directions provides a finer resolution of the identified binding pockets compared to other implementations. This additional information was used to create a new descriptor combining knowledge of shapes with the buriedness of binding-sites. By this means we were able to classify active sites of homologue aldose reductase structures, thereby avoiding the application of sophisticated visual inspections. Results turned out to be promising for shape analyses of closely related enzymes, although 1az1 as the only exception was not properly assigned to the *holo*-conformation class of aldose reductase. This might be due to the fact that this crystal structure carries two mutations within its active site, appreciably changing the shape of the active site.

Pocket analyses revealed a considerable conformational similarity of the active sites of 1eko and 1el3. Although these two proteins originate from different species and share a sequence identity of only 87%, a pronounced adaptation to their common ligand IDD384 could be registered. This circumstance again emphasizes the ability of aldose reductase to react with induced-fit behavior upon ligand binding.

**Figure 11:**
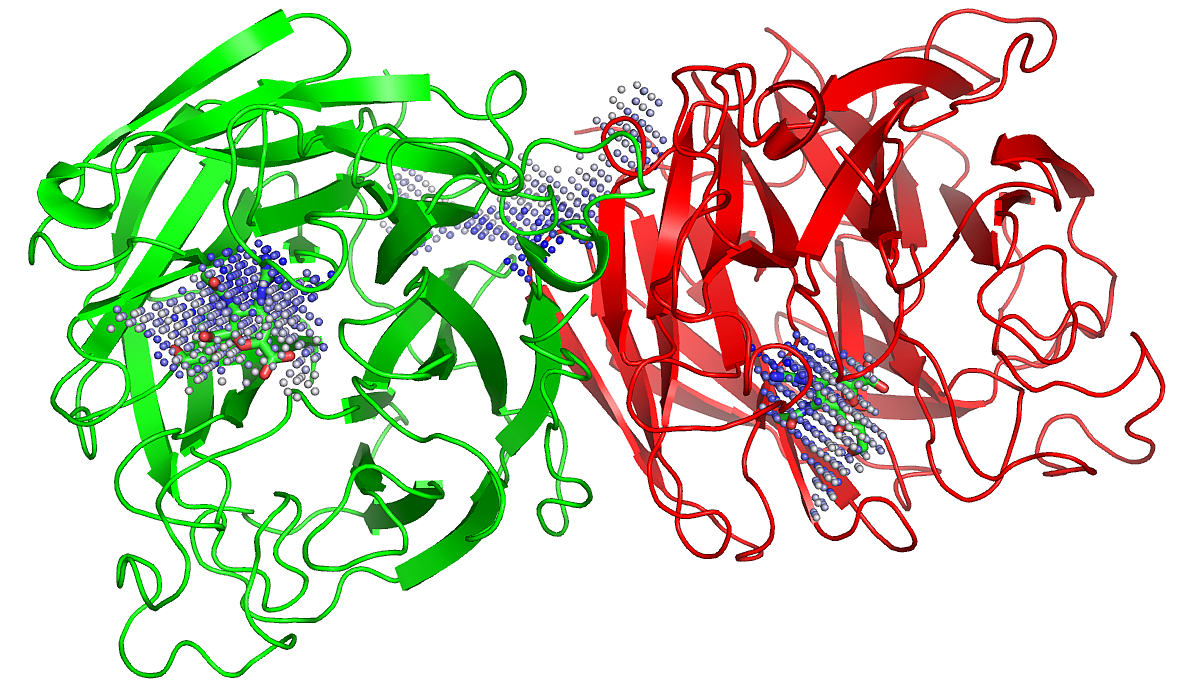
Pocket prediction for influenza virus neuramidase (PDB: 1a4g). A cleft formed between chains A and B is found to be the largest pocket and mistakenly predicted as the actual binding site. The binding sites for the ligands zanamivir (PDB: zmr) are identified as second and third largest pockets.

Best results in terms of prediction success rates were observed when applying PocketPicker to comparably small monomeric proteins (< 5000 atoms). Multimeric proteins composed of identical subunits often form clefts between contact faces that can be mistaken as binding sites (Figure 11).

**Figure 12:**
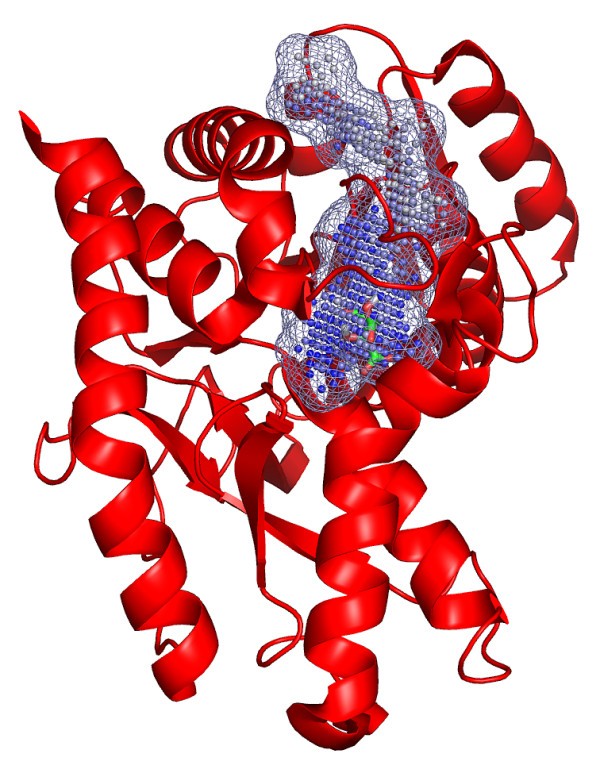
Binding site prediction for malate dehydrogenase (PDB: 2cmd). The ligand citrate (PDB: cit) is situated in the distant end of the elongated pocket (mesh representation) that is suggested as the largest pocket by PocketPicker (blue spheres). Due to the particular shape of the pocket this example is not rated as a correct prediction as the closest ligand atom exceeds the maximal preset distance of 4 Å towards the pocket center.

It is therefore recommended to perform binding site predictions on monomeric structures. Predictions for large proteins (> 8000 atoms) turned out to be difficult as disjunct pockets were sometimes joined by narrow "tunnels" underneath the protein surface. The criterion used to assess the quality of pocket predictions raises further problems that can affect the actual prediction success. Thus, Top1-hits may not be considered as correct predictions for small ligands that reside in the distant end of an elongated pocket and, therefore, exceed the maximum distance of 4 Å towards the geometric pocket center (Figure 12).

For the sake of completeness we tested PocketPicker on a test set of 210 complexes compiled by Huang and Schröder [17]. This test set also contained multimeric structures. Success rates of PocketPicker predictions for Top1- and Top3-hits on this test set were considerably lower than on the set of 48 bound/unbound structures (Table 5). The reduced performance of PocketPicker might be due to the fact that this test set includes a considerable number of large proteins. It has been observed that the active site volume scales with the protein size whereas there is little correlation between protein volume and ligand volume [26]. This circumstance complicates predictions made by PocketPicker as the method is designed to identify ligand binding sites of limited size for shape comparisons.

**Table 5:** Success rates [%] of PocketPicker on a test set of 210 complexes compared to the results published by Huang and Schröder [17].

	**Top 1**	**Top 3**
LIGSITE^csc1^	75	-
LIGSITE^cs^	67	87
LIGSITE	65	85
PocketPicker	59	71
PASS	54	79
SURFNET	42	56

^1^Results of LIGSITE^csc ^are given for the sake of completeness. Note that the comparability to the other methods findings is limited, due to the fact that LIGSITE^csc ^is not a purely geometric approach.

It has been observed by us and others that predicted pockets are often larger than the volume occupied by a ligand [33]. This fact complicates automated shape comparison, because two pockets can possess a similar ligand binding site but have different volumes overall. Future work will be devoted to narrowing the definition of a "pocket" to the actually preferred ligand binding volume. This also includes the introduction of an energy-based approach to complement the geometric algorithm used in PocketPicker.

## Conclusion

We successfully developed and applied the automated pocket detection method PocketPicker to the task of identifying ligand binding sites in proteins, and the task of clustering structurally related binding sites by shape and a buriedness index. It was demonstrated that the search routine of PocketPicker is capable of identifying the active site within a protein structure with a high success rate on a representative test set.

## Availability and requirements

PocketPicker was designed as a plugin for PyMOL [28] (version 0.98). The software is made available *via *our website  together with full documentation.

Project name: PocketPicker;

Project home page: ;

Operating system: Platform independent;

Programming language: Python, PyMOL;

License: modified BSD; a valid license of PyMOL [28] is required.

## Authors' contributions

This work was prepared in the research group of Professor Dr. Gisbert Schneider (Beilstein Endowed Chair For Cheminformatics). The PocketPicker algorithm was developed by Martin Weisel and Ewgenij Proschak. Shape analyses of aldose reductase active sites were carried out by Martin Weisel. This project was based on the idea and realized under the guidance and consultation of Gisbert Schneider.

## Supplementary Material

Additional file 1 Four complexed and three unbound structures (all multimers) were converted into their respective monomeric equivalents in the original test set by deleting chains. Additional Table 1 indicates which chains were deleted to form the monomeric proteins. List of structures converted into their monomeric versions by deleting chains from the original PDB file. Click here for file

Additional file 2 Four complexed and three unbound structures (multimers) were converted into their respective monomeric equivalents in the original test set by deleting chains (see additional Table 1). Results for calculations on the multimeric proteins are presented in additional file 2). Results of PocketPicker calculations on seven multimeric structures contained in the test data set. ^1^Rank of pocket-centers within 4 Å of the considered ligand (brackets indicate hits exceeding the 4 Å criterion). Only the best hit is shown. Dashes indicate that the actual binding site is not found within the five largest predicted pockets. ^2^Distance from the geometric pocket center to the nearest atom in the ligand. ^3^Pocket detection for 1a4j failed, due to PyMOL's inability to align the dimer structure with its corresponding monomeric complex 1igj. Click here for file
